# Impact of a pioneer diabetes camp experience on glycemic control among children and adolescents living with type 1 diabetes in sub-Saharan Africa

**DOI:** 10.1186/s12902-016-0086-x

**Published:** 2016-01-20

**Authors:** Mesmin Y. Dehayem, Rémy Takogue, Siméon-Pierre Choukem, Olivier T. S. Donfack, Jean-Claude Katte, Suzanne Sap, Eugène Sobngwi, Jean-Claude Mbanya

**Affiliations:** Endocrine and Diabetes Service, Yaoundé Central Hospital, PO Box 87, Yaoundé, Cameroon; Health and Human Development Research Group, Douala, Cameroon; Diabetes and Endocrine Unit, Internal Medicine Service, Douala General Hospital, Douala, Cameroon; Department of Internal Medicine and Pediatrics, Faculty of Health Sciences, University of Buea, Buea, Cameroon; Laboratory for Molecular Medicine and Metabolism, Biotechnology Centre, University of Yaoundé I, Yaoundé, Cameroon; Bafoussam Regional Hospital, Bafoussam, Cameroon; Mother and Child Centre, Chantal Biya Foundation, Yaoundé, Cameroon; Faculty of Medicine and Biomedical Sciences, University of Yaoundé I, Yaoundé, Cameroon

**Keywords:** Type 1 diabetes, Children, Diabetes camps, Glycemic control

## Abstract

**Background:**

The metabolic impact of participating in a diabetes camp is little known among children and adolescents living with type 1 diabetes in sub-Saharan Africa. We aimed to assess the changes in glycemic control and insulin doses in a group of children and adolescents living with type 1 diabetes in Cameroon during and after camp attendance.

**Methods:**

During a 5-day camp, we collected data on insulin doses, HbA1c, weight and blood glucose at least six times per day in a group of children and adolescents living with type 1 diabetes. We compared the evolution of these parameters 3 and 12 months after camp.

**Results:**

Thirty-two campers completed the study. The mean age was 19 ± 2 years and the median duration of diabetes was 2 [IQR: 1.8–5] years. The mean HbA1c was 7.9 ± 2.2 % and the mean insulin dose was 49 ± 20 units/day upon arrival at camp. HbA1c dropped by 0.6 % after 12 months (*p* = 0.029). Despite the significant (*p* = 0.04) reduction in insulin dose from 49 ± 20 to 44 ± 18 units/day at the end of camp, hypoglycemic episodes occurred in 26 campers. However, the mean number of hypoglycemic episodes reduced from 1.32 (range: 0–4) on the first day, to 0.54 (range: 0–2) on the last day of camp (*p* = 0.006). Weight increased by 6 kg (*p* = 0.028) between 3 and 12 months after camp, but insulin doses remained unchanged.

**Conclusions:**

Attending camp for children and adolescents living with diabetes is associated with a significant decrease in HbA1c twelve months after camp without changes in insulin doses. Including camps as an integral part of type 1 diabetes management in children and adolescents in sub-Saharan Africa may yield some benefits.

**Trial registration:**

ClinicalTrials.gov NCT02632032. Registered 4 December 2015.

**Electronic supplementary material:**

The online version of this article (doi:10.1186/s12902-016-0086-x) contains supplementary material, which is available to authorized users.

## Background

Therapeutic education is central to the management of diabetes, especially in children and adolescents [1]. To be effective, therapeutic education should be individually and collectively structured aiming to empower patients’ ability to manage their condition and to improve their quality of life [2]. Camps for children and adolescents living with diabetes represent an ideal environment for education [3]. These camps are increasingly becoming popular with nearly 46,000 children and adolescents participating each year worldwide [4]. During camps, the campers receive both theoretical and practical information intended to improve their understanding of diabetes [5]. Meals, insulin doses and physical activity programs are developed in order to help campers to achieve better glycemic control [6].

Several studies have shown the positive impact of camps on the level of knowledge and social welfare of campers as well as their ability to self-support [7–10]. However, their metabolic impact is still debated and very little is known on this subject in Sub-Saharan Africa.

Since October 2010, the “Changing Diabetes in Children” (CDiC) project has structured the management system of diabetes in children and adolescents in Cameroon. Nine specialized clinics have been created all over the country, and the project has organized training sessions for 664 health personnel. Furthermore, campaigns have been organized to raise awareness at local and national level, and a national registry for diabetes in children has also been launched. More importantly, free diabetes care is being offered to all children and adolescents aged 18 years or less at enrolment into the project. The project also organizes two annual camps for children and adolescents living with diabetes. Unlike with previous editions, the fifth camp organized in Yaoundé in July 2013 included adolescents above 18 years. Also, it included only patients from Yaoundé and surrounding areas. This rendered follow-up after camp more feasible. The aim of this study was to assess changes in glycemic control and insulin doses in this group of campers, during the camp, as well as 3 and 12 months after camp.

## Methods

### Participants and setting

In this study, we analyzed data obtained from children and adolescents living with type 1 diabetes who attended the diabetes camp of July 2013 and who came back for follow up at the CDiC clinic of the Yaoundé Central Hospital, 3 and 12 months later.

The CDiC project offers logistics and free medical care to children living with type 1 diabetes in Cameroon, which includes free medical consultations, insulin, syringes, a glucose meter (Accu Check Active®, Roche Diagnostics GmbH, Mannheim, Germany) glucose strips, HbA1c monitoring every 3 months, collective therapeutic education sessions every 3 months and a yearly screening for complications. All children enrolled in the project have a systematic medical visit every 3 months. During this visit, clinical and biological assessments are done, as well as adjustment of treatment where necessary. It also always includes a therapeutic education session delivered by a dedicated specialist nurse. All information obtained is recorded in the patient’s medical record. Many children also consult outside systematic visits to collect material for treatment or in the event of an emergency whether related or not to diabetes. The forms of insulin available in the project are regular insulin (Actrapid®), intermediate-acting insulin (Insulatard®) and pre-mixed insulin (Mixtard 30®). HbA1c is assessed by the in2it™ point-of-care system (Bio-Rad Laboratories, Deeside, UK).

The CDiC project organizes a 5-day camp for about 50 children twice yearly. Participants are selected by the health care personnel in charge of their care, based on the availability of places and on the proximity to their homes. Children aged less than 6 years, those with an acute disease and those with incomplete recovery from a previous illness are not allowed to camp.

### Camp organization

A written informed consent was obtained from parents or guardians before inclusion and a medical insurance was contracted for campers and the camp staff. The camp staff consisted of a pediatric endocrinologist, an adult endocrinologist, 3 general physicians, 4 nurses, a dietician and a sports coach. The leisure program during the camp included educational workshops, games, sporting activities, a visit to an animal reserve and dinner at a local restaurant. Meals offered to the children attending the camp were prepared following the instructions of the dietician.

Treatment protocols and insulin doses of each participant were maintained upon arrival. However, during the camp and before every meal, the dose of insulin to be injected was analyzed and eventually modified by the physician based on the results of self-monitoring of blood glucose, the quantity of carbohydrates to be ingested, and the level of physical activity to be performed. Notwithstanding, the treatment protocols could still be modified for some children who were poorly controlled.

At the end of camp, a prescription was done for every camper and an adjustment of doses was made based on capillary blood glucose. Capillary blood glucose was measured six times a day (before and 2 h after the 3 main daily meals), and as needed (before and after any intense physical activity session, as well as in the event of a symptoms suggestive of hypoglycemia). Finally, all information on each camper (injected insulin doses, capillary blood glucose, HbA1c, weight and eventual malaise) were recorded in a self-monitoring booklet by the camper with supervision of a member of camp staff.

### Post-camp data collection

Campers were later routinely followed-up at the Yaoundé CDiC clinic. Of the 46 patients who attended the camp, only the 32 who came for both 3 and 12 months’ follow-up visits were included in further analyses. We collected data on age, gender, duration of diabetes, duration of follow-up in the CDIC project, weight, insulin regimen and insulin doses at the beginning, at 3 and 12 months after camp, HbA1c at the beginning, at 3 and at 12 months after camp, and the daily number of hypoglycemic episodes during camp. Good glycemic control was defined as HbA1c <7.5 % and hypoglycemia as capillary blood glucose < 70 mg/dl.

### Data analysis

Data were analyzed using the Statistical Package for Social Sciences version 12 (SPSS Inc. Chicago, IL USA). Results are presented as mean and standard deviation or median [interquartile range] for continuous variables and as count (percentage) for discrete variables. We compared proportions by the Mc Nemar’s test for paired data, means by repeated measure ANOVA, paired *t* test or independent *t* test where appropriate and medians by the Wilcoxon rank sum test. Where necessary, continuous variables were categorized using the median as cutoff. Analysis of factors associated with HbA1c level was restricted to 3 months because of limitation associated with data availability. A *P*-value < 0.05 was used to characterize statistically significant results.

### Ethical considerations

Prior to enrolment in the CDiC project, a written informed consent form was signed by parents or guardians authorizing the CDiC project in Cameroon to use the data obtained for research. The CDiC project has also received approval from the National Ethics Committee of Cameroon (Authorization Nο 271/CNE/SE/2011) to carry out research from data obtained in the project. This study received an approval by the CDiC project steering committee.

## Results

### Baseline characteristics

Thirty-two campers were re-evaluated 3 and 12 months after camp. None of them had ever attended a camp before. Table 1 describes the characteristics of the study population (See also Additional file 1: Data of the 32 campers, and Additional file 2: Gender distribution, median age and location of campers). Most patients were above 18 years of age, 13 were aged 12–18 years, and only one patient was below 12 years. The majority (25/32) of campers were treated with multiple daily injections of insulin. There was no significant difference at baseline between the 32 campers included in analysis, and the 14 with incomplete follow-up data who were excluded, with regards to the mean age, sex-ratio, median duration of diabetes and median duration of follow-up in the CDiC project.

**Table 1:** Baseline characteristics of campers and median number clinic visits and capillary blood glucose/day 3 months after camp (*N* = 32)

Variable	Value
Gender (female/male)	13/19
Mean age (years)	19 ± 2
Mean weight (kg)	63 ± 12
Mean HbA1c (%)	7.9 ± 2.2
Mean insulin dose (units/day)	47 ± 20
Median [IQR] duration of diabetes (years)	2 [1.8–5]
Median [IQR] duration of follow-up in the CDiC project (years)	1.8 [0.7–2.5]
Median [range] number of visits to the clinic 3 months after camp	2 [0–4]
Median [range] number of glycaemia/day 3 months after camp	3 [0–3]
*Distribution of children according to the number of insulin injections per day n (%) N* = *32*	
Two	5 (16)
Three	27 (84)

*IQR* interquartile range

### Outcomes during and after camp

The mean HbA1c decreased by 0.6 % after 12 months (*p* = 0.029), and the number of patients who reached HbA1c level < 7.5 % increased from 47 % at the beginning of camp to 69 % 12 months later. The mean daily insulin doses decreased by 6.4 % (*p* = 0.04) between the beginning and the end of camp, and remained unchanged 12 months after camp (Table 2). Out of the 32 campers, insulin doses were reduced in 18 (56 %), increased in 6 (19 %) and maintained in 8 (25 %). The average weight gain was 6 kg between 3 and 12 months after camp (*p* = 0.028) (Table 2). During the 5 day-camp, 752 capillary glucose tests were performed in the 32 campers and 100 episodes of hypoglycemia were recorded in 26 of them, with a maximum of 10 episodes in one child. No severe hypoglycemia was recorded. The mean number of hypoglycemic episodes reduced significantly (*p* = 0.006) the last day [0.54 (range: 0–2)] of camp when compared to the first day [1.32 (range: 0–4)] (Table 2).

**Table 2:** Outcomes at the end, and at 3 and 12 months after camp (*N* = 32)

Parameters	Beginning of camp	End of camp	3 months after camp	12 months after camp	Overall p value
Mean HbA1c (%)	7.9 ± 2.2^α^	-	7.6 ± 2.2	7.3 ± 1.9^α^	0.088^*^
Mean Insulin dose (unit/day)	47 ± 20^β^	44 ± 18^β^	44 ± 21	45 ± 21	0.39^*^
Mean Weight (kg)	63 ± 12	-	59 ± 16^δ^	65 ± 12^δ^	0.05^*^
HbA1c < 7.5 %, n/N (%)	15/32 (46.9)	-	14/32 (43.7)	22/32 (68.8)	0.18^Ω^
Reduction of HbA1c, n/N (%)^a^	/	/	18/32 (56)	21/32 (66)	0.53
Reduction of insulin doses, n/N (%)^b^	/	18/32 (56)	15/32 (47)	/	0.59

*repeated measures ANOVA

^α^ *p* = 0.029 between beginning of camp and 12 months after camp (paired *t* test)

^β^ *p* = 0.04 between beginning and end of camp (paired *t* test)

^δ^ *p* = 0.028 between 3 months after camp and 12 months after camp (paired *t* test)

^Ω^Proportion between the beginning and the end of camp (Mc Nemar test)

^a^Reduction of HbA1c% if (HbA1c% at the beginning of camp – HbA1c% 3 or 12 months after camp) > 0

^b^Reduction of insulin dose if (insulin dose at the beginning of camp – insulin dose at the end of camp or 3 months after camp) > 0

### Factors associated with post-camp HbA1c levels

Diabetes duration less than 2 years (*p* = 0.041), visiting the clinic more than 2 times during the 3 months of post-camp follow up (*p* = 0.045), and monitoring blood glucose more than twice daily (*p* = 0.005) were significantly associated with lower HbA1c levels (Table 3). We found no association between gender, age and time spent in the CDiC project, and HbA1c levels (Table 3).

**Table 3:** Factors associated with HbA1c level 3 months after camp

	Median HbA1c [IQR]	*p*-value*
Gender		
Male (*n* = 19)	8.1 [6.9–8.6]	0.11
Female (*n* = 13)	6.7 [5.8–7.9]	
Age		
< 19 years (*n* = 14)	6.9 [5.3–8.4]	0.12
≥ 19 years (*n* = 18)	8.1 [6.7–8.7]	
Duration of diabetes		
≤ 2 years (*n* = 17)	5.9 [5.3–8.4]	0.041
> 2 years (*n* = 15)	8.1 [7.8–8.5]	
Duration in CDiC project		
≤ 1.8 years (*n* = 16)	6.4 [5.6–8.4]	0.073
> 1.8 years (*n* = 16)	8.1 [6.8–9.1]	
Number of visits at the clinic		
≤ 2 (*n* = 21)	8.1 [6.8–8.5]	0.045
> 2 (*n* = 11)	5.9 [5–8]	
Number of glycaemia/day		
≤ 2/day (*n* = 15)	8.4 [7.9–9.5]	0.005
> 2/day (*n* = 17)	6.0 [5.8–8]	

*Wilcoxon rank sum test

## Discussion

In this study, we observed a significant decrease in HbA1c in a group of sub-Saharan African children and adolescents living with type 1 diabetes 12 months after their first participation in a diabetes camp. We also found a concomitant reduction in exogenous insulin needs and an increase in their weight.

The impact of camps on the improvement of glycemic control is controversial. Some studies have shown an improvement in glycemic control including a significant reduction in HbA1c and other glycated proteins after camp [11, 14–17], whereas others have not [10–13].

A Thai study reported an initial significant drop in HbA1c three months after camp, followed by an increase at six months. In that study, the authors noted a positive correlation between the number of daily capillary blood glucose, and the decrease in HbA1c [16]. Other studies have reported a greater decrease in HbA1c after camp in children with the worst pre-camp glycemic control as well as in those regularly attending diabetes camps [11]. According to the authors the improvement in glycemic control after camp would be attributed to a better knowledge acquisition and diabetes management skills during camp [4, 10, 11, 14–16]. Unlike all the other studies, the decrease in HbA1c in our study was more gradual and continuous, and was significant only after 12 months.

We observed an association between the reduction of HbA1c and the following factors: the number of capillary blood glucose measurements done by the camper, the number of hospital visits after camp and the duration of diabetes. There was no significant immediate impact of the camp on glycemic control. However, a significant improvement was observed only after a long term follow up. The non-significant decrease in HbA1c at three months probably reflects the fact that initial HbA1c was not very high (7.9 %). The continuing decline in HbA1c at 12 months in patients who carried out more capillary blood glucose measurements or attended more hospital visits indicates the importance of reinforcing the knowledge and skills acquired during camp on the subsequent visits. Noteworthy, the same medical and nurse personnel provided care to the campers both in camps and in the hospital, and therefore were probably more knowledgeable regarding social, psychological and emotional aspects of the campers. This likely served to further strengthen their education. The significant weight at three months could be explained by an increase in physical activity and diet control efforts in the campers after camp.

Hypoglycemia was very common at the beginning of camp. The frequency was higher than that reported by Maslow and Lobato in a literature review on diabetes camps [11]. This was probably related to the increase in physical activity and the healthier dietary measures adapted to the nutritional needs of each camper while maintaining the pre-camp insulin doses. To reduce the frequency of hypoglycemia during camp, organizers usually monitor and reduce the usual insulin doses of campers by 10–30 % upon arrival [5, 10, 15, 18]. The reduction in insulin doses alongside an appropriate dietary plan permitted the significant reduction in the frequency of hypoglycemia, which went down from 1.3 episodes per camper per day at the beginning to 0.5 at the end of the camp.

Our study has some limitations. The sample size was small, although comparable to that of the majority of quality prospective studies [5, 10, 15, 17]. The initial number of campers was limited to ensure better monitoring and also to avoid accidents. In addition, 14 participants were not reassessed after three months or 12 months and were excluded from the final analysis which further reduced the size of our sample. The height of campers at 3 and 12 months was not available for most participants and could therefore limit the anthropometric evaluation. As another limitation, we used a point-of-care device, which, although reliable, does not give HbA1c values above 14 %; this may have underestimated the overall mean HbA1c. However, this was very limited because only one value was above 14 % at each time point. The majority of campers lived in the city of Yaoundé and could easily gain access to the clinic for monitoring after the camp; the results may therefore not be generalizable to the country and sub-Saharan Africa. Despite these limitations, this study is, to the best of our knowledge the first to assess the metabolic impact of camps in children and adolescents with diabetes in sub-Saharan Africa and has provided key messages. Skills acquired during camp may not explain alone the improvements of glycemic control after camp, but may be the triggering factor.

## Conclusions

Our study indicates that camps can have a positive and lasting metabolic impact on glycemic control in young patients living with type 1 diabetes in sub-Saharan Africa. This does not only require good organizational plan of the camp, but also the frequent reassessment of these same campers to reinforce and consolidate the acquired knowledge. The increase in the frequency of hypoglycemia recorded at the beginning of the camp should lead to the systematic reduction of insulin doses on arrival at camp, especially in well-controlled campers.

## Additional files

Additional file 1:
**Data of the 32 campers.** (XLSX 19 kb) Additional file 2:
**Gender distribution, median age and location of campers.** (DOCX 12 kb)
