# Supplementary material for: Impact of a pioneer diabetes camp experience on glycemic control among children and adolescents living with type 1 diabetes in sub-Saharan Africa
Source: BMC Endocr Disord. 2016 Jan 20;16:5. doi: 10.1186/s12902-016-0086-x (PMC4721200; doi:10.1186/s12902-016-0086-x)
Supplement: Additional file 2: — Gender distribution, median age and location of campers. (DOCX 12 kb) [file 12902_2016_86_MOESM2_ESM.docx]

**Some baseline characteristics of campers (N = 32)**

| **Variable** | **Value** |
| --- | --- |
| **Gender (female/male)** | 13/19 |
| **Age (years), median (range)** | 19 (9-22) |
| **Location** |  |
| Yaoundé | - |
| Akonolinga | 117 km from Yaoundé |
| Bafia | 124 km from Yaoundé |
| Mbalmayo | 52 km from Yaoundé |
| Monatelé | 76 km from Yaoundé |
| Ndom | 124 km from Yaoundé |
